# Enhanced Separation Performance of Hierarchically Porous Membranes Fabricated via the Combination of Crystallization Template and Foaming

**DOI:** 10.3390/polym14235160

**Published:** 2022-11-27

**Authors:** Jiahui Shi, Jiahai Zhou, Donglei Fan, Taotao Lin, Jiayao Wang, Jiaqi Zhao, Avner Ronen, Minggang Li, Jichun You

**Affiliations:** 1 Key Laboratory of Organosilicon Chemistry and Material Technology, Ministry of Education, College of Material, Chemistry and Chemical Engineering, Hangzhou Normal University, Hangzhou 311121, China; 2 Zhejiang Chuanhua Chemical Group Co., Ltd., Hangzhou 311215, China; 3 State Key Laboratory of Polymer Physics and Chemistry, Changchun Institute of Applied Chemistry, Chinese Academy of Sciences, Changchun 130022, China; 4 Jacob Blaustein Inst Desert Res, Zuckerberg Inst Water Res, Sede Boqer Campus, Ben Gurion Univ Negev, Beer Sheva IL-84990, Israel

**Keywords:** hierarchically porous membrane, PVDF, foaming, crystallization template, separation

## Abstract

In this work, poly (vinylidene fluoride) (PVDF) hierarchically porous membranes (HPMs) with isolated large pores and continuous narrow nano-pores have been fabricated from its blend with poly (methyl methacrylate) (PMMA) based on the combination of crystallization template with chemical or supercritical CO_2_ foaming. On the one hand, the decomposition of azodicarbonamide (ADC, chemical foaming agent) or the release of CO_2_ can produce isolated large pores. On the other hand, PMMA is expelled during the isothermal crystallization of PVDF in their miscible blend, yielding narrow nano-pores upon etching with a selective solvent. In the case of supercritical CO_2_, the attained PVDF HPMs fail to improve separation performance because of the compact wall of isolated-large-pore and consequent poor connectivity of hierarchical pores. In the case of ADC, the optimal HPM exhibits much higher flux (up to 20 times) without any loss of selectivity compared with the reference only with nano-pores. The enhanced permeability can be attributed to the shorter diffusion length and lower diffusion barrier from isolated large pores, while the comparable selectivity is determined by narrow nano-pores in THE matrix.

## 1. Introduction

Separation membranes have been widely used in various fields [1,2,3,4,5]. During separation, selectivity and permeability are key parameters that can be assessed by rejection ratio and flux, respectively. It is relatively simple to improve the selectivity by decreasing pore size. The smaller pores, however, correspond to poor permeability and vice versa. This is well-known trade-off effect in the field of separation, and recent decades have witnessed significant efforts to stop this effect [6,7]. Among them, hierarchically porous membranes (HPMs) with pores in at least two scales have been regarded as one of the most perspective strategies for practical applications, such as water–oil separation, ion separation and dye separation [8,9,10]. Obviously, the selectivity depends on the matrix’s pore size in hierarchically porous membranes. The existence of porous structure in the other scale in HPMs contributes to shorter diffusion length and lower diffusion barrier, and the additional diffusion channels, both of which are beneficial for the enhancement of permeability.

Two types of hierarchically porous membranes are available. In Type I, large pores connect, accounting for the main diffusion channels on which small pores have been fabricated [11,12]. The main diffusion channels determine the selectivity. The existence of small pores can enhance the permeability of HPMs. In Type II, narrow nano-pores act as a matrix while isolated large pores are distributed in it [13,14]. Obviously, Type II is highly in demand in terms of separation because it corresponds to much higher selectivity compared to Type I. In our previous work, this kind of HPM was obtained with the help of a dual template in a ternary blend of poly (vinylidene fluoride), poly (methyl methacrylate) and poly (L-lactic acid) (i.e., PVDF/PMMA/PLLA). 

Phase separation produces the matrix of a PVDF/PMMA mixed phase and islands of the PLLA phase. In the miscible blend of PVDF/PMMA, PMMA is expelled during the crystallization of PVDF. Upon etching with chloroform, both PMMA and PLLA can be removed, yielding PVDF HPMs with pores in and nanometers. Relative to the reference with only nano-pores, the attained HPMs exhibit much higher flux and comparable rejection ratio [15]. The key to the further improvement of HPMs flux is a higher content of isolated large pores. Unfortunately, a further increase in the PLLA weight fraction in the ternary blend leads to phase inversion and a continuous state of PLLA in the phase-separated structures (i.e., PVDF/PMMA islands in the PLLA matrix) [15].

**Figure 1:**
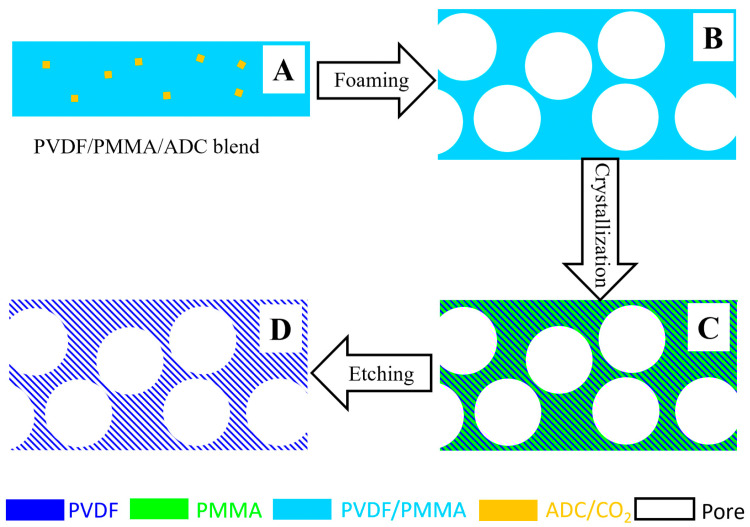
Illustration of fabrication of PVDF HPMs via the combination of crystallization template and foaming. The specimen of hot-pressed PVDF/PMMA/ADC blend (**A**), upon foaming (**B**), isothermal crystallization (**C**) and etching (**D**).

Therefore, in this work, we propose the fabrication of HPMs (Type II) based on combining a crystallization template and foaming (Figure 1). The blend of PVDF/PMMA is employed to prepare narrow nano-pores, while supercritical carbon dioxide (CO_2_) or azodicarbonamide (ADC) is adopted as a forming agent to fabricate the pores in microns (Figure 1A). In the case of supercritical CO_2_, the PVDF/PMMA blend is kept in a CO_2_ atmosphere at a high temperature, followed by the release of gas. During chemical foaming, the hot-pressed PVDF/PMMA/ADC blend is heated above the foaming temperature of ADC. The release of CO_2_ or the decomposition of ADC at this temperature generates isolated large pores in the PVDF/PMMA mixed phase (Figure 1B) [16,17,18].

During the subsequent isothermal crystallization of PVDF, PMMA will be expelled, accounting for the bi-continuous structures of PVDF and PMMA [19]. Upon etching, narrow nano-pores can be obtained (Figure 1C). The combination of isolated large pores (from ADC or CO_2_) and nano-pores in the matrix (from PMMA) produces PVDF HPMs (Figure 1D) in which the former and the latter determine higher permeability and superior selectivity, respectively. Their synergism contributes to enhanced permeability and comparable selectivity relative to the reference with only nano-pores.

## 2. Experimental

### 2.1. Materials

PVDF (KF850, *M*_w_ = 209,000 g/mol, PDI = 2.0) and PMMA (*M*_w_ = 35,000 g/mol, PDI = 2.0) were provided by Kureha Chemicals (Tokyo, Japan) and Sigma-Aldrich (St. Louis, MI, USA), respectively. Azodicarbonamide (ADC) and ethanol were produced by Sinopharm Chemical Reagent (Shanghai, China). Chloroform was provided by Lingfeng Chemical Reagent (Shanghai, China). Albumin from bovine serum (BSA, purity 98%) was purchased from J&K Scientific.

### 2.2. Sample Preparation

The PVDF/PMMA blend, with a weight ratio of 50/50, was prepared by melting-blending in the internal mixer (Haake Polylab QC, Thermo Fisher Scientific, Waltham, MA, USA) at 200 °C. In the case of supercritical CO_2_, the batch foaming method was used in which PVDF/PMMA blend was kept in a CO_2_ atmosphere at 150 °C and 12 MPa for 4 h, followed by the release of CO_2_. During chemical foaming, PVDF/PMMA/ADC blends were prepared by adding ADC (1 wt%, 2 wt%, 3 wt%) to PVDF/PMMA blend and mixed at 175 °C for 3 min. The melting-blended mixture was hot-pressed into a film (thickness = 600 μm) in a flat vulcanizer at 180 °C and 25 MPa. After cooling to room temperature, the hot-pressed films were placed into a 230 °C oven for various periods. The foamed specimens (from CO_2_ or ADC) were quickly transferred to a 150 °C oven for full crystallization of PVDF. They were then transferred to the Soxhlet extraction device and extracted by chloroform for 48 h. Then, the specimen was immersed in ethanol for 2 h and transferred to deionized water, followed by freeze-drying at −100 °C for 24 h.

### 2.3. Characterization and Instrumentation

The pore morphology of HPMs was detected by field-emission scanning electron microscope (FESEM, Hitachi S-4800, Tokyo, Japan) after sprayed with gold by ion sputtering apparatus (Hitachi E-1010, Tokyo, Japan). The pore sizes were measured by Nano Measurer software (Department of Chemistry, Fudan University, Shanghai, China, Version: 1.2.5) through the analysis of SEM images. Nano Measurer is an open-source software widely used by researchers [20,21,22]. Nano Measurer can statistically analyze pore numbers, size and other dimensions of SEM/TEM images and can process and export pore size distribution data. The thermogravimetric analysis confirmed the removal of PMMA and ADC in PVDF/PMMA/ADC blends (TGA, TA Q500, New Castle, PA, USA). A 5 mg specimen was placed into the TGA tray and heated from 30 °C to 650 °C at a heating rate of 10 °C/min in a nitrogen atmosphere. With the help of a homemade filtration device, deionized water and a BSA solution were selected to assess the permeability and selectivity according to the method described in our previous work [14].

The pure water flux of each membrane was evaluated by a self-made filtration setup (a schematic illustration of separation setup is shown in Figure S2) under a driving pressure of 1 bar, and each membrane was tested at least five times. The flux is measured after the membranes separate the liquid for 5 min. The water volume and valid membrane area in this work are 5 mL and 3.14 cm^2^, respectively. The flux (*J*) was calculated according to Equation (1),

(1) $$J=\frac{V}{At∆p}$$

where *V*, *A*, *t* and Δ*p* are the water volume, valid membrane area, testing time and driving pressure, respectively. The rejection ratio of the hierarchically porous membranes was evaluated by taking BSA solution (2 g/L, dissolved in 0.01 mol/L phosphate buffer saline) as the model system based on Equation (2),

(2) $$R\left(\%\right)=\left(1-\frac{C_{p}}{C_{f}}\times 100\right)$$

where *C_p_* and *C_f_* were the BSA concentrations of permeate and feed solutions, respectively. The concentrations were measured by UV–vis spectrophotometer at a fixed wavelength of 280 nm.

## 3. Results and Discussion

**Figure 2:**
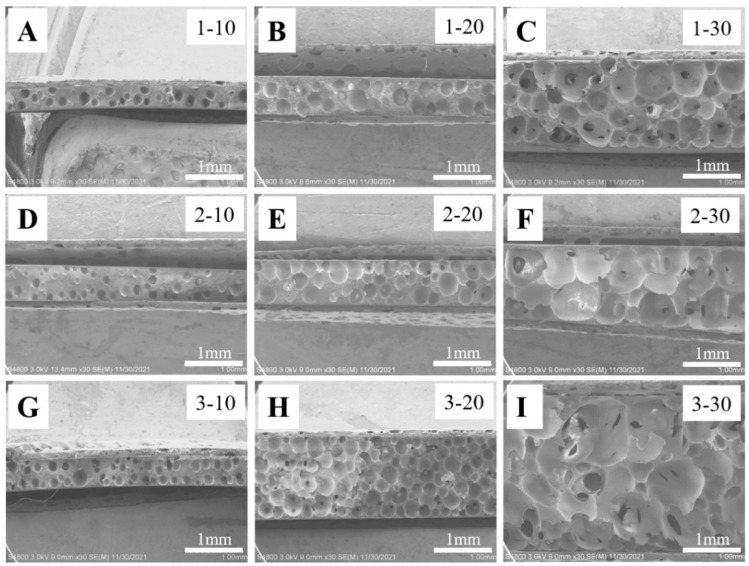
SEM images of the cross–sections of foamed PVDF/PMMA blend films with the indicated ADC content (1% in (**A**–**C**)), (2% in (**D**–**F**)), (3% in (**G**–**I**)) and foaming time (10 min in (**A**,**D**,**G**)), (20 min in (**B**,**E**,**H**)), (30 min in (**C**,**F**,**I**)).

First of all, our attention was paid to ADC foaming. For simplicity, the specimen with *a*% ADC foaming for the *b* min is named a-b, sic passim. On the fracture surface of specimens before foaming (Figure S1), ADC has no aggregation even when its weight fraction reaches 3%, indicating the excellent dispersity of ADC in the PVDF/PMMA blend. Moreover, no porous structure can be observed after hot-pressing at 180 °C since this temperature is below the decomposition temperature of ADC (~230 °C, determined by TGA, shown in the following parts). When our specimens are heated to 230 °C and kept for a certain period at this temperature, ADC in the blend decomposes into nitrogen (N_2_), carbon monoxide (CO), carbon dioxide (CO_2_), ammonia (NH_3_) and other kinds of gases [23,24,25,26]. They are generated in PVDF/PMMA blends continuously in a molten state, yielding cellular porous structures with the size of hundreds of microns in the PVDF/PMMA matrix (Figure 2). 

**Figure 3:**
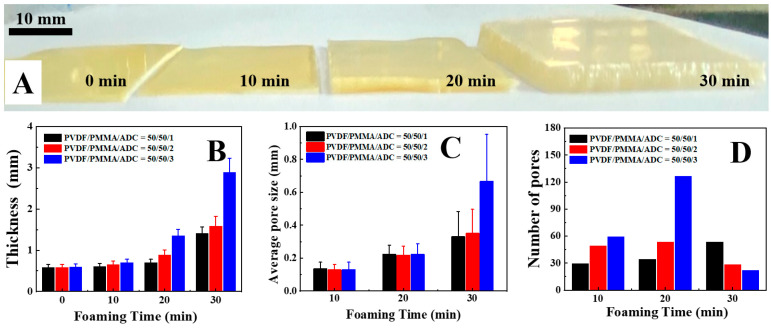
Photos (**A**) of a foamed PVDF/PMMA blend and its foaming time dependence on thickness (**B**), pore size (**C**) and pore number (**D**).

During the chemical foaming process of ADC, there are significant evolutions of film thickness, pore size and pore number, which have been discussed in detail as follows. Firstly, the length and width of our specimen remain almost constant while their thicknesses increase significantly. The photos and the statistical results are shown in Figure 3A (3% ADC) and Figure 3B, respectively. In the original specimen, the thickness is 0.6 mm. It reaches 0.7 mm, 1.4 mm, and 2.9 mm upon foaming for 10 min, 20 min and 30 min, respectively (Figure 3B). Secondly, the size of the foamed pores is controlled by the weight fraction of ADC and foaming time (Figure 2). The higher ADC content (specimens of 1–10, 2–10 and 3–10) and an extended foaming period (specimens of 1–10 to 1–30, 2–10 to 2–30, and 3–10 to 3–30) produce bigger cellular pores. The foaming time and ADC content dependencies on the pore size are shown in Figure 3C. For instance, the pore diameter increases from 0.14 mm to 0.33 mm upon foaming for 10 min and 30 min (1% ADC). After sufficient foaming, it is 0.33 mm and 0.67 mm in 1% and 3% ADC, respectively. Finally, the number of pores in the corresponding SEM images (Figure 2) depends crucially on ADC content and foaming time (Figure 3D).

In the case of lower ADC content (1% ADC), the pore number on the fracture surface (specimen 1–10, 1–20 and 1–30, shown in Figure 2A–C) increases monotonously upon further foaming. In the results of the 2% (Figure 2D–F) and 3% (Figure 2G–I) ADC; however, these large pores connect with each other when the foaming time reaches 30 min, leading to the merger of neighboring pores, higher magnitudes of pore diameter (Figure 3C), and a consequent sudden drop in pore number (specimen of 2–30 and 3–30). According to the discussion above, it is relatively simple to fabricate cellular pores with the help of ADC foaming. Both pore size and pore number can be tailored by means of ADC content and foaming time. It is noteworthy that excessive foaming (higher ADC content and longer foaming time) produces large connected pores, leading to interpenetrated diffusion channels with a higher pore diameter.

**Figure 4:**
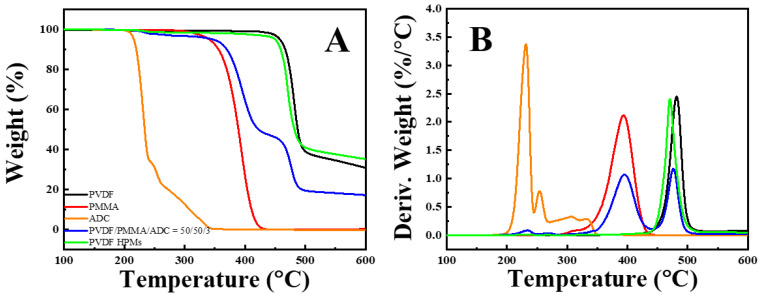
TGA (**A**) and DTG curves (**B**) of PVDF, PMMA, ADC, PVDF/PMMA/ADC (50/50/3) blends and PVDF HPMs.

In the foamed specimens, chloroform was employed as a selective solvent to extract PMMA. A thermogravimetric analysis (TGA) test was performed to validate its complete removal, and the results are shown in Figure 4 [27]. The degradation behaviors of neat PVDF, PMMA and ADC were investigated in the index peaks in the blend specimen (blue curve in Figure 4). The main degradation peaks of PVDF (black curve) and PMMA (red curve) are located at 481.1 °C and 393.7 °C, respectively. There are several degradation peaks (231.2 °C, 254.3 °C, 306.8 °C and 332.3 °C) in the results of ADC in Figure 4B, suggesting the different chemical intermediate steps [28]. The first one appears at 231.2 °C. This result makes it clear that the ADC remains stable during hot-pressing of PVDF/PMMA/ADC at 180 °C. It decomposes into various gases only when it is heated above 230 °C. In the PVDF/PMMA/ADC blend, the peaks at 233.4 °C, 394.8 °C and 476.5 °C can be ascribed to the degradation of ADC, PMMA and PVDF, respectively. The area fractions of these peaks are undoubtedly a necessary consequence of weight ratios in the blend. 

Upon etching with chloroform, the attained PVDF HPMs exhibit similar degradation behaviors with neat PVDF (Figure 4A). The main degradation peak locates at 470.3 °C, close to neat PVDF. The slight decrease in degradation temperature can be attributed to the porous structures in PVDF HPMs. The disappearance of the PMMA degradation peak in the green curve in Figure 4B confirms that the PMMA were removed successfully from its blend with PVDF. Furthermore, there is a peak (located at 233.4 °C) in the results of the HPMs in Figure 4B. It originates from the degradation of residual ADC. It is reasonable to conclude that most ADC were removed since the area fraction of the peak is below 1%. The chemical foaming and removal of PMMA by means of etching produce round pores and nano-pores, respectively. Therefore, the PVDF HPMs with isolated large pores connected through narrow pores in nanometers have been fabricated successfully, which will be validated by means of SEM in the following parts.

**Figure 5:**
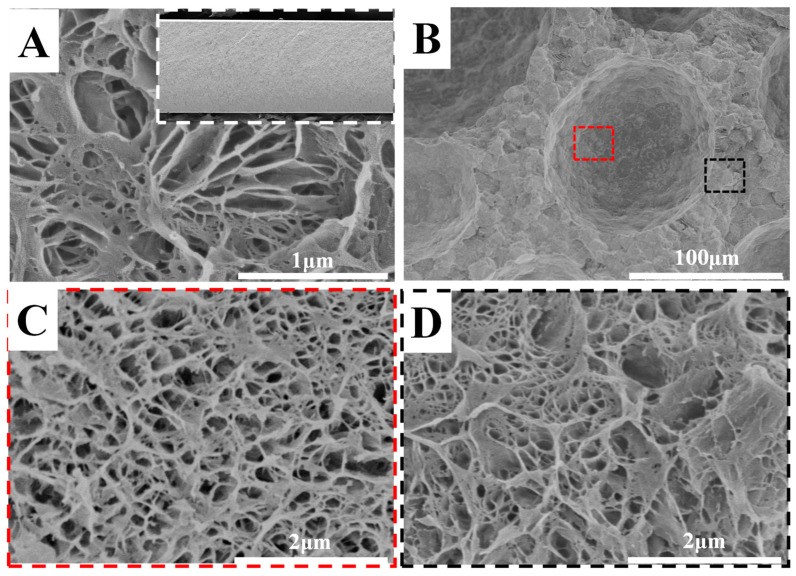
SEM images of the fracture surface of the reference (**A**), PVDF HPMs (2–20) (**B**) and images with higher magnifications (**C**,**D**). Inset in (**A**) shows the whole cross-section of reference. The red and black dash boxes represent the magnified images at the indicated position.

Figure 5 shows SEM images of resultant PVDF HPMs and the reference with only nano-pores. In Figure 5A, the fracture surface looks “homogeneous” (inset), which is completely different from the results in Figure 2. In the SEM images of higher magnification, there are narrow porous structures in the size of hundreds of nanometers. In the PVDF HPMs, we paid attention to the specimen from 2–20 because it shows balanced permeability and selectivity, which will be discussed in the following. In Figure 5B, some large cellular pores on the cross-section are obvious, and their diameter was in the hundreds of microns. These pores act as islands, distributing in the matrix with nano-pores. SEM images with higher magnifications were taken at the indicated position to show the fine structures clearly. In Figure 5C,D, there are narrow nano-pores on the wall of isolated large pores as well as in the matrix. These nano-pores resemble closely with the reported porous structures from the crystallization template [19]. The porous structures on the pore wall endow isolated large pores and narrow nano-pores with excellent connectivity, producing interpenetrated diffusion channels for the fluid through the whole porous membrane.

According to the discussion above, we can describe the formation of hierarchically porous structures in the PVDF/PMMA/ADC blend as follows. After melting-mixing, the ternary blend was hot-pressed at 180 °C. ADC remains stable since this temperature is below its decomposition temperature (Figure 4B). Heated to 230 °C, ADC decomposes into kinds of gases, yielding cellular isolated large pores in hundreds of microns (Figure 2). Then, the specimen was kept in an oven at 150 °C for full crystallization of PVDF. In the miscible PVDF/PMMA blend, the crystallization of PVDF takes place because this temperature is between its glass transition temperature (roughly at −35 °C) and melting temperature (~170 °C). During this process, PMMA is expelled and segregates into the inter-lamellar or inter-fibrillar regions, resulting in the bi-continuous structures of PVDF and PMMA. Upon etching with chloroform, the selective solvent for PMMA, PMMA can be removed, yielding narrow nano-pores. This scenario has good agreement with the formation of porous structures based on crystallization template, which was discussed in detail in our previous works [19].

**Figure 6:**
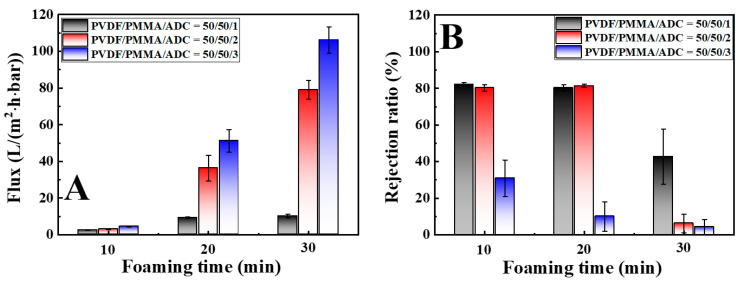
Flux of pure water (**A**) and rejection ratio in the separation of BSA solution (**B**) of various PVDF HPMs.

To test the separation performance of the attained PVDF HPMs with isolated large pores connected through narrow nano-pores, pure water and BSA solution were taken as model systems. In membrane separation, both porous structures and the wettability of the adopted liquid on membrane surface play vital roles [28,29,30,31,32]. In this work, however, all HPMs exhibit similar surface porous structures (Figure S3) and water contact angles (90°–96°). The separation performance, therefore, is mainly dominated by isolated large pores in membranes. In reference to only the narrow nano-pores, the flux is 1.8 L/m^2^·h bar. Figure 6A shows the fluxes of PVDF HPMs obtained from specimens with various ADC filling amounts and foaming times. Our attention was paid to the following points. Firstly, the flux exhibits much higher magnitudes upon foaming than the reference, indicating the positive effect of isolated large pores on permeability [14]. Secondly, higher content of ADC results in higher flux. Upon foaming for 20 min, PVDF HPMs from 1–20, 2–20 and 3–20 correspond to the flux of 9.3, 36.3 and 51.2 L/m^2^·h·bar, respectively. Finally, the flux increases with increasing foaming time. For instance, it is 3.1 L/m^2^·h·bar in the specimen from 2–10. Upon further foaming for 20 and 30 min, the fluxes reach 36.3 and 78.9 L/m^2^·h·bar, respectively. The significant increase in flux, even in the PVDF HPMs with much thicker membranes (up to 4.8 times shown in Figure 2 and Figure 3B), further identifies the key role of isolated large pores in improving permeability.

It is noteworthy that the water flux depends on the thickness according to Darcy’s law. The magnitudes of flux can be improved by decreasing membrane thickness. The selectivity of PVDF HPMs has been assessed by filtrating BSA solution [33,34,35,36,37]. As shown in Figure 6B, the specimens from 1–10, 1–20, 2–10 and 2–20 exhibit a rejection ratio of ~80%. This value is comparable with that in reference (81.5%). In this case, the narrow nano-pores in a matrix determine the selectivity of HPMs [14]. The excessive increases of ADC content or foaming time lead to a much lower rejection ratio (4%–30%). The sudden selectivity drop suffers from the interpenetrated large pores from ADC foaming (Figure 2). In other words, these large pores connect, producing a continuous diffusion channel with bigger diameters. As a result of balanced permeability and selectivity, the PVDF HPM from the specimen of 2–20 can be regarded as the optimal specimen in which the narrow nano-pores in the matrix determine the higher selectivity while the large pores from ADC foaming act as islands, contributing to shorter diffusion length. The isolated large pores, narrow nano-pores in the matrix and excellent connectivity between them contribute to the interpenetrated diffusion channels running through the pores in two scales. During the transportation of fluid through the whole membrane, a higher content of isolated large pores can further shorten the diffusion length and reduce the diffusion barrier, which is the reason for the significant improvement in permeability (up to 20 times) [14].

According to the discussion above, chemical foaming by ADC works well in the fabrication of HPMs. The resultant membranes exhibit enhanced permeability and comparable selectivity compared with the reference. Excessive foaming leads to connected large pores and a sudden drop in rejection ratio during the separation of BSA. To fabricate HPMs with a higher content of isolated large pores, supercritical CO_2_ foaming has been employed. Specimens of PVDF/PMMA blend with a weight ratio of 50/50 were kept in a CO_2_ atmosphere for 4 h for saturation, followed by gas release.

**Figure 7:**
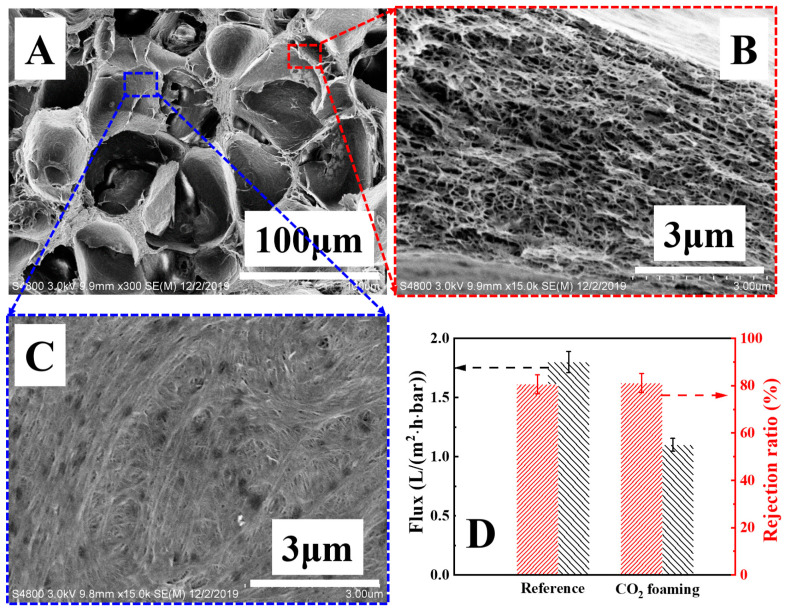
SEM images (**A**–**C**) of PVDF HPMs upon foaming with supercritical CO_2_ and separation performance (**D**). The red and blue boxes represent the magnified images at the indicated position.

After the full crystallization of PVDF, PMMA was removed by means of Soxhlet extraction with chloroform. As shown in Figure 7A, there are uniform cellular pores with a size of tens of microns on the fracture surface. The pores, coming from the release of CO_2_, do not connect. In other words, the pore wall among neighboring cells isolates them, yielding a matrix (red regions) and isolated large pores (blue regions). In SEM images with higher magnifications of the former (Figure 7B), narrow nano-pores distribute in the matrix. They are typical porous structures from crystallization templates [19]. On the wall of large pores; however, there is no porous structure, only a rough surface (Figure 7C). The compact pore wall and the absence of nano-pores can be interrupted as follows. During saturation at high temperatures and high pressure, CO_2_ distributes in the PVDF/PMMA blend homogeneously. Upon release, the CO_2_ bubbles nucleate and grow. Due to the similar chemical structures and favorable interaction between CO_2_ and PMMA (relative to PVDF), the PMMA migrates to CO_2_. The enrichment of PMMA near the CO_2_ bubbles leads to the PMMA layer and PMMA depletion sub-layer (i.e., neighboring PVDF layer). Upon etching with chloroform, the PMMA layer has been removed. The neighboring PVDF layer is exposed to air, accounting for compact pore walls. This assumption has been validated by varying foaming temperature and saturation pressure, in which there are always compact pore walls (data not shown here). 

The separation performances of PVDF HPMs from CO_2_ have been assessed. As shown in Figure 7D, the water flux of reference is ~1.8 L/m^2^·h·bar. This value decreases to 1.1 L/m^2^·h·bar in the case of PVDF HPMs from CO_2_. The lower magnitude of flux suffers from more tortuous diffusion channels and longer diffusion pathways since compact pore walls and poor connectivity between cellular pores (from CO_2_) and narrow nano-pores (from PMMA) prevent fluid entrance into isolated large pores. In the separation of the BSA solution, the rejection ratios of reference and PVDF HPMs are comparable. In sum, the PVDF HPMs from the CO_2_ fail to enhance the separation performance.

## 4. Conclusions

Along with the crystallization template in the miscible PVDF/PMMA blend, both supercritical CO_2_ and ADC have been employed and work well in the fabrication of HPMs. On the one hand, the release of CO_2_ or decomposition of ADC produces isolated large pores. On the other hand, PMMA was expelled during the crystallization of PVDF. Narrow nano-pores can be obtained upon etching with chloroform. The combination of them contributes to the successful fabrication of PVDF HPMs. In the case of supercritical CO_2_, PVDF HPMs fail to enhance separation performance because of the compact wall of isolated large pores and poor connectivity of pores in two scales. PVDF HPMs from ADC, however, exhibit much higher flux (up to 20 times) and comparable rejection ratio relative to reference with only nano-pores. Such a significant improvement in permeability can be attributed to the shorter diffusion length from the isolated large pores, while the comparable selectivity arises from the nano-pores in the matrix of the HPMs. Our results provide a solution to improve the content of isolated large pores in HPMs, and, thus, the trade-off effect in their separation. The resultant HPMs also exhibit potential applications, including, but not limited to, the loading of functional materials in isolated large pores in future applications.

## Data Availability

The data presented in this study are available on request from the corresponding author.

## Supplementary Materials

The following supporting information can be downloaded at: https://www.mdpi.com/article/10.3390/polym14235160/s1, Figure S1: The SEM images with low (A–C) and high magnifications (D–F) of PVDF/PMMA/ADC hot-pressed films filled with 1 wt% (A,D), 2 wt% (B,E) and 3 wt% (C,F) ADC before foaming; Figure S2: Home-made filtration device; Figure S3: SEM image of the surface of PVDF HPMs with the indicated ADC filling amount and foaming time.
