# Supplementary material for: Enhanced Separation Performance of Hierarchically Porous Membranes Fabricated via the Combination of Crystallization Template and Foaming
Source: Polymers (Basel). 2022 Nov 27;14(23):5160. doi: 10.3390/polym14235160 (PMC9736639; doi:10.3390/polym14235160)
Supplement: Supplementary file 1 [file polymers-14-05160-s001.zip › polymers-2019311-supplementary.pdf]

Supporting information for

Enhanced Separation Performance of Hierarchically Porous Membranes Fabricated via the Combination of Crystallization Template and Foaming

Jiahui Shi [1], Jiahai Zhou [2], Donglei Fan [3], Taotao Lin [1], Jiayao Wang [1], Jiaqi Zhao [1], Avner Ronen [4], Minggang Li [3*], Jichun You [1*]

1 *Key Laboratory of Organosilicon Chemistry and Material Technology, Ministry of Education, College of Material, Chemistry and Chemical Engineering, Hangzhou Normal University, Hangzhou, 311121, China*

2 *Zhejiang Chuanhua Chemical Group Co., Ltd., Hangzhou 311215, China*

3 *State Key Laboratory of Polymer Physics and Chemistry, Changchun Institute of Applied Chemistry, Chinese Academy of Sciences, Changchun 130022, China*

4 *Jacob Blaustein Inst Desert Res, Zuckerberg Inst Water Res, Sede Boqer Campus, Ben Gurion Univ Negev, Beer Sheva IL-84990, Israel*

* *Correspondence: author: lmg@ciac.ac.cn (M.L.); you@hznu.edu.cn (J.Y.)*

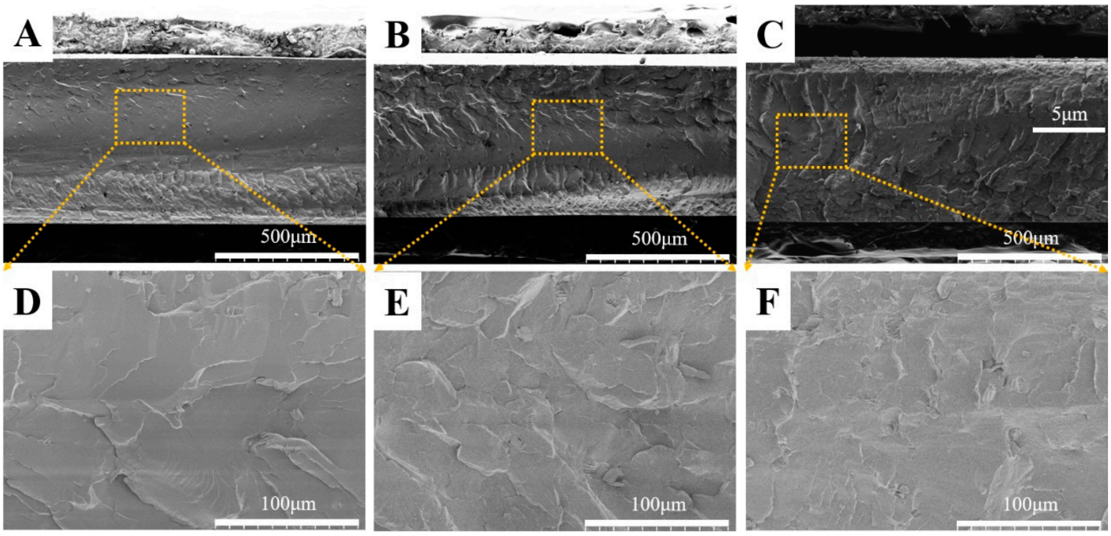

Figure S1. The SEM images with low (A-C) and high magnifications (D-F) of PVDF/PMMA/ADC hot-pressed films filled with 1 *wt*% (A, D), 2 *wt*% (B, E) and 3 *wt*% (C, F) ADC before foaming.

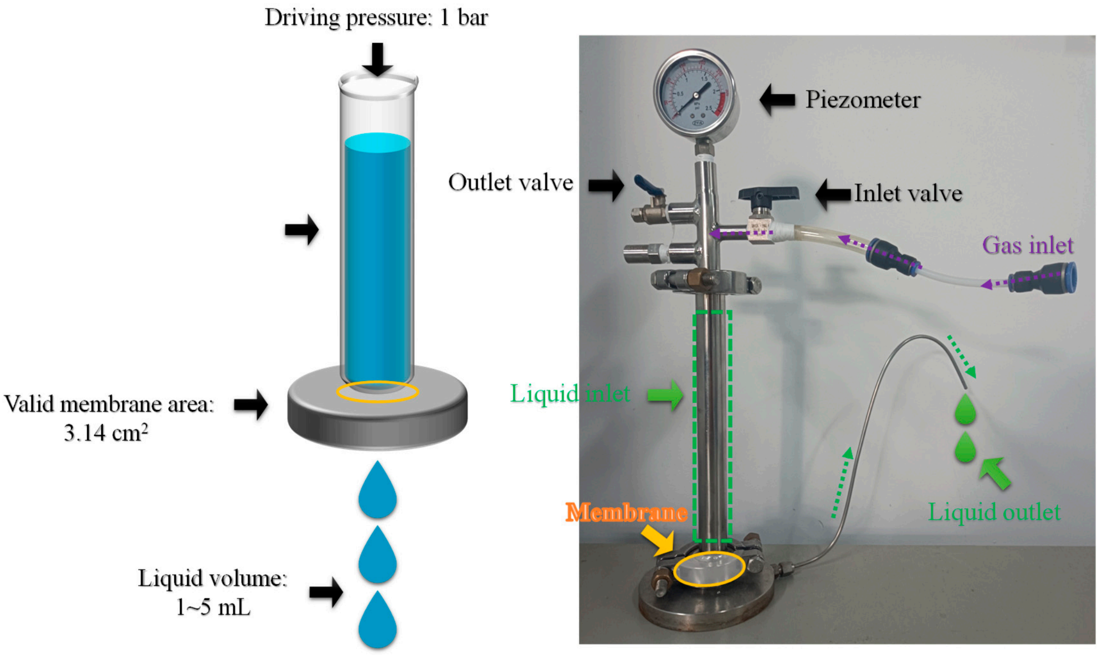

Figure S2. Home-made filtration device

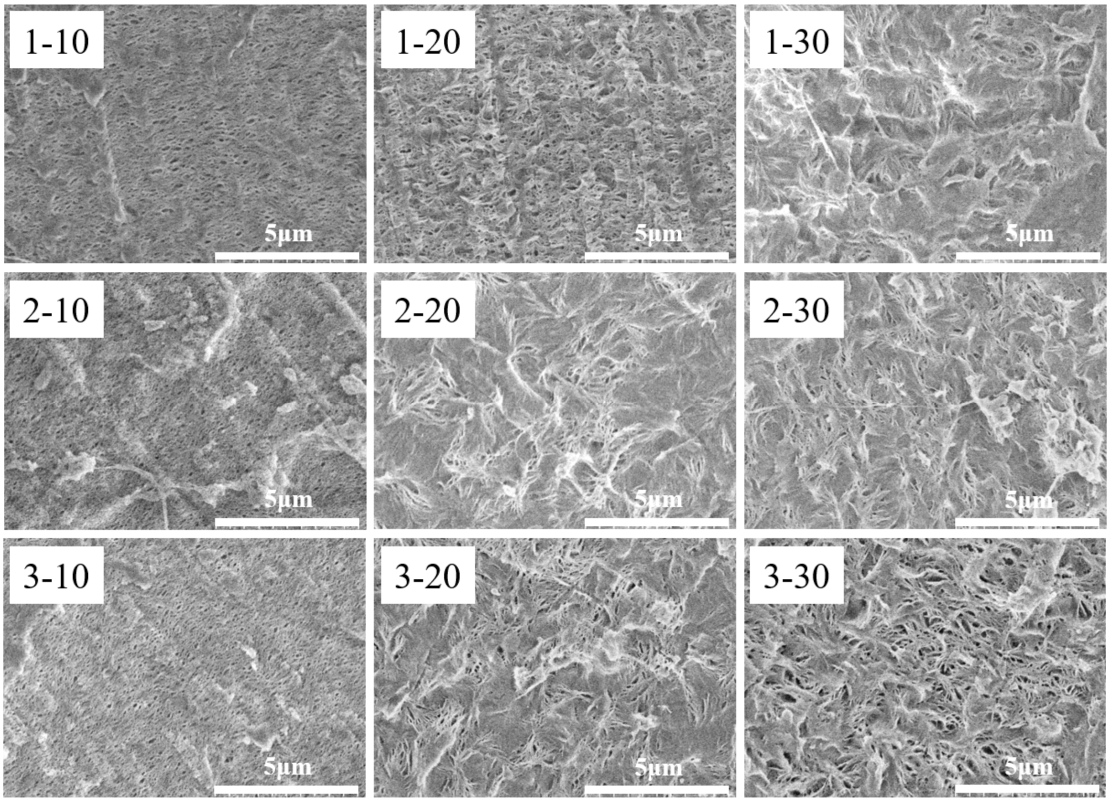

Figure S3. SEM image of the surface of PVDF HPMs with the indicated ADC filling amount and foaming time
